# Diversity of microbial communities potentially involved in mercury methylation in rice paddies surrounding typical mercury mining areas in China

**DOI:** 10.1002/mbo3.577

**Published:** 2018-03-12

**Authors:** Xin Liu, Anzhou Ma, Guoqiang Zhuang, Xuliang Zhuang

**Affiliations:** ^1^ University of Sciences and Technology of China Hefei China; ^2^ CAS, Research Center for Eco‐Environmental Sciences Key Laboratory of Environmental Biotechnology Chinese Academy of Sciences Beijing China; ^3^ College of Resources and Environment University of Chinese Academy of Sciences Beijing China

**Keywords:** diversity, *hgcA*, methylmercury, microbial community, rice paddy

## Abstract

Mercury can be a serious hazard to human health, especially in paddy soils surrounding mining areas. In this study, mercury (Hg)‐methylating microbes with the potential biomarker gene *hgcA* were obtained from 45 paddy soil samples in mercury mining areas in Fenghuang, Wanshan, and Xunyang. In different areas, the abundance of the *hgcA* gene was affected by different environmental factors, including organic matter, pH, total carbon content, total nitrogen content, and total mercury content. Phylogenetic analysis showed that *hgcA* microbes in paddy soils were potentially members of the phyla Proteobacteria, Euryarchaeota, Chloroflexi, and two unnamed groups. Canonical correspondence analysis showed that pH and organic matter impacted the *hgcA* gene diversity and the microbial community structures in paddy soils. The identification of Hg‐methylating microbes may be crucial for understanding mercury methylation/demethylation processes, which would be helpful in assessing the risk of methylmercury contamination in the food chain.

## 1 INTRODUCTION

As a global pollutant, mercury can be transported long distances in gaseous form through atmospheric circulation (Lindqvist et al., 1991) and can be converted to neurotoxic methylmercury (MeHg) via anaerobic microbial actions (Hu et al., 2013). Methylmercury, which bioaccumulates in the food chain, is the most toxic form of mercury (Stein, Cohen, & Winer, 1996) and can affect the central nervous system by crossing the blood–brain barrier.

Recent studies have demonstrated that human MeHg exposure in China is primarily caused by rice consumption (Meng et al., 2014; Rothenberg, Windham‐Myers, & Creswell, 2014; Zhang, Feng, Larssen, Qiu, & Vogt, 2010), rather than by the consumption of fish (Clarkson, 1993). Paddy soil, due to alternating wet and dry cycles, is a typical ephemeral wetland that is conductive to the accumulation of MeHg (Rothenberg & Feng, 2012). Accordingly, the production of MeHg in paddy soils by anaerobic microorganisms has become a major public health issue (Meng et al., 2011). The microbial composition in soil is primarily determined by land‐use patterns (Wu et al., 2017), and the composition of microbial communities in soil may depend on the type of stress that is present (Tardy et al., 2014). In paddy soils surrounding mercury mining areas, mercury pollution (including total Hg and MeHg) significantly affects the bacterial community structure and the bacterial abundance is significantly correlated with the organic matter (OM) content (Liu, Wang, Zheng, Zhang, & He, 2014), although the presence of genes and mechanisms that are related to these conditions are still unknown. Many microbes are sensitive to environmental changes and can respond rapidly to these changes, and these microbes are regarded as efficient bioindicators of soil quality (Nielsen et al., 2002).

Mercury (Hg)‐methylating pathways have been primarily identified in sulfate‐reducing bacteria (SRB) (Gilmour, Henry, & Mitchell, 1992), iron‐reducing bacteria (IRB) (Fleming, Mack, Green, & Nelson, 2006; Kerin et al., 2006; Yu et al., 2012), and methanogens (Hamelin, Amyot, Barkay, Wang, & Planas, 2011; Wood, Kennedy, & Rosen, 1968; Yu, Reinfelder, Hines, & Barkay, 2013). The identification of a two‐gene cluster (consisting of *hgcA* and *hgcB*) in Hg‐methylating microbes (Parks et al., 2013) has resulted in the identification of many microorganisms with the capacity to methylate mercury other than SRB, IRB, and methanogens, including syntrophic Proteobacteria, Firmicutes, and Euryarchaeota (Gilmour et al., 2013; Parks et al., 2013). It has been argued that mercury methylation is associated with the reductive acetyl‐coenzyme A (acetyl‐CoA) Wood–Ljungdahl carbon fixation pathway (Choi, Chase, & Bartha, 1994). The gene *hgcA* encodes a putative corrinoid protein that is involved in the acetyl‐CoA pathway, and *hgcB*, which is adjacent to *hgcA*, encodes a 2[4Fe‐4S] ferredoxin that is required for turnover (Parks et al., 2013; Poulain & Barkay, 2013). Because the *hgcAB* gene pair is widely distributed among Hg‐methylating microbes, and the *hgcA* gene product may provide a methyl group to methylate inorganic mercury (Podar et al., 2015), *hgcA* can serve as a mercury methylation biomarker for evaluating potential Hg‐methylating microbes in soil. Moreover, the relationships between microbial community composition and environmental factors, with respect to mercury methylation potential in the environment, could be investigated using functional genes (Schaefer, Kronberg, Morel, & Skyllberg, 2014).

The aims of this study were to assess the abundance and diversity of the *hgcA* gene in paddy soils surrounding three typical mercury mining areas in China, and to investigate the potential changes in the diversity and distribution patterns of *hgcA*‐containing microorganisms associated with different environmental factors.

## 2 MATERIALS AND METHODS

### 2.1 Site description and sampling

**Figure 1:**
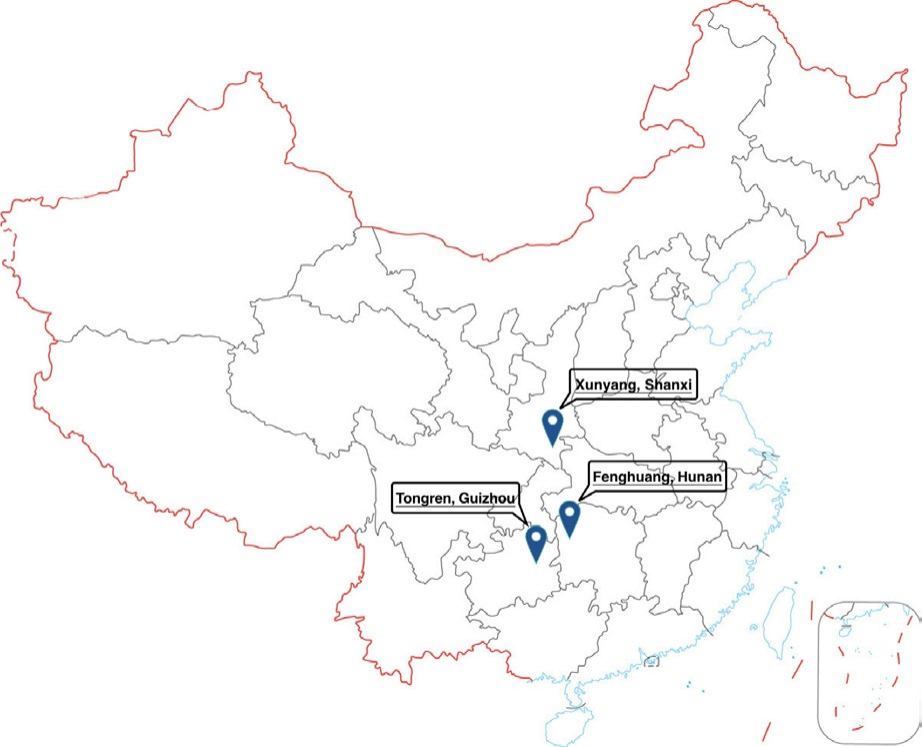
Locations of the three research areas in China

Soil profiles were collected from three typical mercury mining sites, the Wanshan, Fenghuang, and Xunyang mercury mining areas (Figure 1). The Wanshan area is located in Tongren Country, Guizhou Province (109°12'E, 27°31'N) and was ranked the largest mercury‐producing region in China. The mine was initiated during the Qin Dynasty (221 B.C.) and stopped production in 2001. Abandoned mercury mines have an abundance of mining and processing waste (Qiu et al., 2008; Zhang, Feng, Larssen, Shang, et al., 2010). The mine waste calcine piles have released mercury, which has significantly contaminated the local environment (Li et al., 2008; Qiu, Feng, Wang, Fu, & Shang, 2009). The Fenghuang area is located in Hunan Province (110°02'E, 28°01'N) and is well known for the presence of low‐temperature hydrothermal mercury sphalerite (Li, Zhang, Yang, & Li, 2012). The Xunyang area is located in Shanxi Province (109°25'E, 33°06'N) and remains an active mercury mine in China. The primary ore mined in Xunyang is cinnabar, and mercury and antimony ores are found as accessory minerals (Qiu, Feng, Meng, Sommar, & Gu, 2012; Zhang, Jin, Lu, & Zhang, 2009; Zhang, Tang, Chen, Leng, & Zhao, 2014).

Research samples were collected from nine sites (referred to as g1, g2, and g3 in Guizhou; h1, h2, and h3 in Hunan; and s1, s2, and s3 in Shanxi). A total of 45 soil samples (five parallel replicates from each site) were taken at a soil depth of approximately 20 cm. All samples were stored in an ice box for transit back to the laboratory. For each soil sample, a subsample was air‐dried for an analysis of soil physicochemical properties, and a second subsample was freeze‐dried for nucleic acid extraction.

### 2.2 Soil analytical methods

Soil pH was measured at an air‐dried soil‐to‐water ratio of 1:2.5 using an electrode method with a pH conductor (Mettler‐Toledo International Inc., Switzerland) (ISO, 2005). The total carbon (TC) and total nitrogen (TN) in soil samples was determined by elemental analysis (Multi N/C 3100, Analytik Jena, Germany) (Jimenez & Ladha, 1993). Soil OM was measured with the K_2_Cr_2_O_7_ oxidation method (Schulte, 1995). The soil ammonium nitrogen (NH_4_^+^‐N) and nitrate nitrogen (NO_3_^−^‐N) were assessed by leaching the soil with a 0.01 mol/L CaCl_2_ solution at a soil‐to‐water ratio of 1:10 and was measured with a continuous flow analyzer (SAN++, Skalar, Holland) (Zhu et al., 2011). The sulfate concentration was measured via barium sulfate turbidity (Washington, Warren, & Karlson, 1972).

To measure the total Hg content, 1 g of soil was digested with HNO_3_‐HCl (10 ml; 4:1, vol/vol) at 105°C for 2 hr, and then 0.5 ml of 0.2 N BrCl was added to oxidize all forms of mercury to divalent mercury. After filtering the solution, 0.1 ml of SnCl_2_ (200 g/L) was added to reduce the divalent mercury to mercury, after which 2 ml of the mixture was decanted and mixed with deionized water to a volume of 40 ml, which was analyzed with a MERX automated total mercury analytical system (Brooks Rand Instruments, USA) according to USEPA Methods 1631 (Chen et al., 2012; USEPA, 2002). Samples were analyzed for MeHg with KBr‐CuSO_4_/solvent and KOH‐methanol/solvent extraction, and MeHg levels were determined using the MERX system following the USEPA Methods 1630 (Wang, Tam, He, & Ye, 2015; USEPA, 2001).

### 2.3 Soil DNA extraction

Soil microbial DNA was extracted from 0.5 g soil samples using a FastDNA^TM^ SPIN kit for soil (MP Laboratory, USA) following the manufacturer's protocol. The concentration and quality of the DNA yields were determined with a ND‐1000 UV‐Vis spectrophotometer (NanoDrop Technologies, USA).

### 2.4 Amplification of the *hgcA* gene and quantitative PCR analysis

The *hgcA* gene was amplified with the following primer pair: forward primer (*hgcA*_261F, 5′‐CGGCATCAAYGTCTGGTGYGC‐3′) and reverse primer (*hgcA*_912R, 5′‐GGTGTAGGGGGTGCAGCCSGTRWARKT‐3′). The forward primer targeted region encoding the highly conserved cap helix of the corrinoid iron–sulfur protein, and the amplified product consisted of 65% of the *hgcA* gene. The primers were designed to be biased toward Deltaproteobacteria (Schaefer et al., 2014). The 25‐μl PCR cocktail contained 12.5 μl of premix (TaKaRa Bio Inc., Japan), 1 μl of each primer (10 μmol/L), 2 μl of DNA template, and 9.5 μl of distilled water. The thermocycler program was as follows: 95°C for 5 min, followed by 30 cycles of 94°C for 1 min, 55°C for 1 min, and 72°C for 1 min, with a final extension at 72°C for 10 min.

An iCycler iQ5 thermocycler (Bio‐Rad) was used for amplification and quantification of the abundance of the *hgcA* gene. Primer pairs for qPCR were as follows: 515F, 5′‐GTGCCAGCMGCCGCGGTAA‐3′ and 806R, 5′‐GGACTACHVGGGTWTCTAAT‐3′; and *hgcA*_261F and *hgcA*_912R (described above). All reactions were performed in a 25‐μl PCR cocktail consisting of 12.5 μl of SYBR Premix Ex Taq^™^ (TaKaRa Bio Inc., Japan), 0.5 μl of each primer (10 μmol/L), 1 μl of 10‐fold diluted DNA template, and 10.5 μl of distilled water. The thermal cycling parameters were as follows: 95°C for 5 min, followed by 40 cycles of 94°C for 30 s, 54°C for 30 s, and 72°C for 1 min. The number of gene copies was calculated by the iCycler iQ Real‐Time Detection System Software (Bio‐Rad Laboratories) using the standard curve method for absolute quantification. To prepare absolute standards, the concentration of plasmids with 16s rDNA or *hgcA* genes was measured with a ND‐1000 UV‐Vis spectrophotometer and converted to copy number using the molecular weight of the DNA or RNA. Standard curves were generated with the threshold cycle (Ct) of nine serial dilutions of plasmid templates (10^9^–10^1^ copies). To convert the DNA copy number present in 1 μl of 10‐fold diluted DNA template to the number of DNA copies in 1 g dry soil, the value obtained by qPCR was multiplied by 400. Each set of qPCR reactions was carried out in triplicate.

### 2.5 Construction of *hgcA* gene clone libraries

In total, nine sites were selected to construct clone libraries. PCR products were ligated to a pGEM‐T Easy Vector (Promega, USA) and transformed into *Escherichia coli* B*M*DH5α cells. Approximately 200 positive clones from each library were randomly selected and sequenced with the M13F/R primers. Sequences were grouped into operational taxonomic units (OTUs) with the program mothur (Schloss et al., 2009) using a similarity threshold of 80% (Schloss & Westcott, 2011; DeSantis et al., 2007).

### 2.6 Phylogenetic analyses

We compared the obtained *hgcA* gene sequences to entries in the NCBI database with the Basic Local Alignment Search Tool (BLAST). Phylogenetic analyses of the taxonomic diversity of fragmented metagenomic *hgcA* sequences were performed using MEGA version 6.0, and neighbor‐joining trees were generated based on the Kimura two‐parameter distance with 1,000 replicates to generate bootstrap values.

### 2.7 Statistical analysis

Statistical analysis was done using IBM SPSS Statistics version 13 (SPSS Inc., Chicago, IL, USA). The data in this research were normally distributed, Kendall's tau‐b correlation analyses among soil parameters were subjected to correlation analysis, and one‐way analysis of variance (ANOVA) was used to assess differences. A Venn diagram illustrating the similarity of the microbial communities in paddy soils from different mercury mining areas was generated. The alpha diversity of *hgcA* genes was determined with mothur software. Canonical correspondence analysis (CCA) was conducted to assess the relationship between detected microbial species and environmental factors.

### 2.8 GenBank accession numbers

The sequence data from OTUs were deposited into GenBank with accession numbers MF168617 to MF168794.

## 3 RESULTS AND DISCUSSION

### 3.1 Soil properties

The concentrations of soil factors in different mining areas are shown in Table 1. The total mercury concentration in different mercury mining areas ranged from 2.09 to 70.65 mg/kg (except for site h1), much higher than what is allowed under the environmental quality of soil inorganic pollutants second‐class standard in China (Ministry of Environmental Protection China, 2008). Long‐term environmental exposure to mercury results in persistent damage to the renal, immune, and central nervous systems in humans. While the extent of the risk to the majority of the population from exposure to existing environmental mercury sources appears to be limited, this conclusion is based on an incomplete database. Thus, it is generally agreed that exposure to various forms of mercury should be minimized (Holmes, James, & Levy, 2009). The MeHg content at different mercury mining areas was significantly correlated with different soil factors, and the Pearson's correlation coefficient was used to describe the correlation between Hg/MeHg content and environmental factors. In the Wanshan mercury mining area, there was a significant positive correlation between the total mercury and methylmercury concentrations in paddy soils (τ = 0.879, *p *<* *.01), which is consistent with the chemical form of mercury affecting the rate of the production of mercury methyl (Benoit, Gilmour, & Mason, 2001). The only environmental factor that had positive correlation with the total mercury content (τ = 0.826, *p *<* *.01) and MeHg content (τ = 0.795, *p *<* *.01) was pH. In the Fenghuang mercury mining area, a significant correlation between environmental factors and the total mercury content was not observed. In the Xunyang mercury mining area, the MeHg content was negatively correlated with OM (τ = −0.696, *p *<* *.01) and total carbon content (τ = −0.979, *p *<* *.01). Most researchers believe that the total mercury concentration is closely related to the OM content, because mercury and OM combine to form complexes (Lindberg & Harriss, 1974). Of course, the combining of mercury and OM is subject to many other factors, such as the degradation and mineralization of OM (Gray & Hines, 2009), the concentration and presence of inorganic mercury, and the type and activity of the SRB (Macalady, Mack, Nelson, & Scow, 2000) and sulfate ion concentration (Branfireun, Roulet, Kelly, & Rudd, 1999). These findings are in agreement with previous studies (Gu et al., 2011; Zhu, Han, & Wu, 2017), indicating that OM and pH had a high impact on MeHg production. The concentration of MeHg in soil samples is the net result of a dynamic equilibrium between Hg methylation, MeHg demethylation, and the reduction of Hg^2+^ to Hg (Bravo et al., 2016).

**Table 1:** Chemical properties of paddy soils in Hg mining areas

Sample	MeHg (μg·kg^−1^)	Total Hg (mg·kg^−1^)	pH	OM (g·kg^−1^)	SO_4_^2−^ (g·kg^−1^)	Total C (g·kg^−1^)	Total N (g·kg^−1^)	NO_3_^−^ (mg·kg^−1^)	NH_4_^+^ (mg·kg^−1^)
g1	4.28 ± 2.42	38.77 ± 12.63	7.66 ± 0.04	19.74 ± 2.17	0.06 ± 0.02	23.23 ± 5.06	1.19 ± 0.14	3.02 ± 0.62	0.13 ± 0.08
g2	3.91 ± 1.04	30.58 ± 6.36	7.65 ± 0.08	24.02 ± 2.06	0.19 ± 0.10	27.48 ± 8.25	1.45 ± 0.12	4.72 ± 2.03	0.22 ± 0.12
g3	0.44 ± 0.20	10.48 ± 0.92	6.61 ± 0.26	19.50 ± 2.12	0.13 ± 0.01	20.33 ± 3.37	1.17 ± 0.12	4.81 ± 2.25	0.37 ± 0.15
h1	1.56 ± 0.51	0.24 ± 0.08	7.45 ± 0.10	14.41 ± 1.51	0.07 ± 0.02	23.59 ± 6.86	0.86 ± 0.08	0.81 ± 0.33	0.54 ± 0.45
h2	0.25 ± 0.14	2.09 ± 0.85	7.20 ± 0.17	21.81 ± 1.81	0.17 ± 0.11	30.41 ± 19.28	1.41 ± 0.19	2.36 ± 0.91	0.20 ± 0.10
h3	1.37 ± 0.62	10.03 ± 6.09	7.69 ± 0.08	21.52 ± 3.56	0.19 ± 0.08	57.28 ± 13.29	1.30 ± 0.21	1.89 ± 0.28	0.14 ± 0.09
s1	0.18 ± 0.07	70.65 ± 9.27	8.09 ± 0.03	47.56 ± 0.56	0.35 ± 0.16	59.01 ± 1.51	2.76 ± 0.22	2.17 ± 0.10	0.70 ± 0.13
s2	2.43 ± 0.52	38.60 ± 24.33	8.20 ± 0.06	24.02 ± 1.67	0.27 ± 0.07	25.40 ± 2.85	1.32 ± 0.07	1.78 ± 0.49	0.44 ± 0.16
s3	3.04 ± 0.16	5.80 ± 0.97	6.39 ± 0.07	36.25 ± 0.41	0.19 ± 0.07	13.01 ± 0.41	2.07 ± 0.14	1.70 ± 0.61	0.81 ± 0.33

Hg, mercury; MeHg, methylmercury; OM, organic matter.

### 3.2 Abundance of the *hgcA* gene

**Figure 2:**
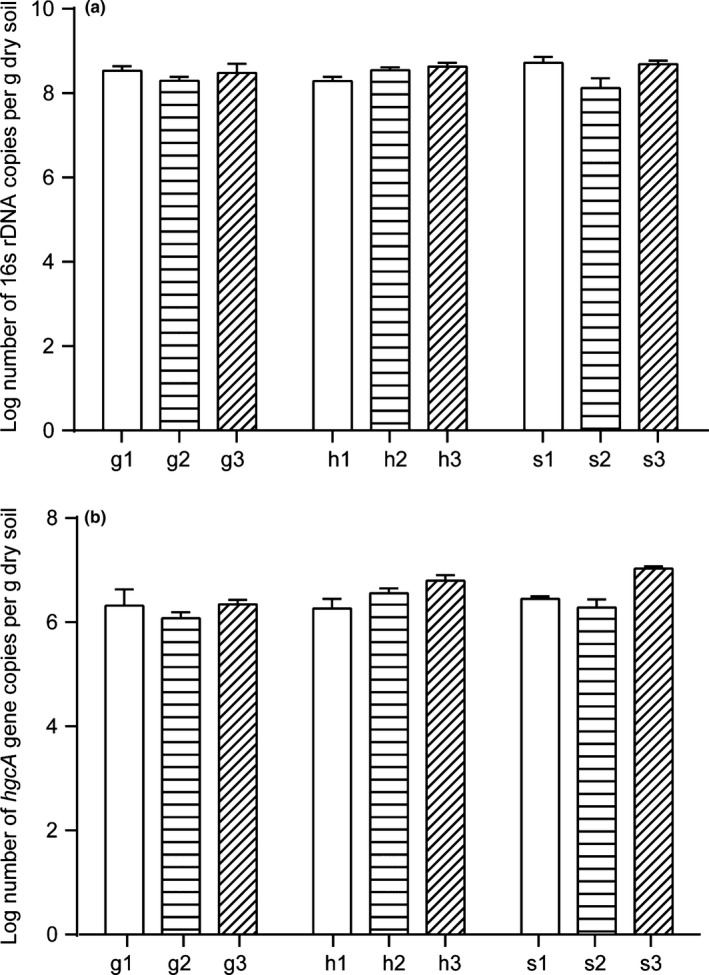
Abundance of 16s rDNA or *hgcA* genes in paddy soils at three Hg mining areas (g1, g2, and g3; h1, h2, and h3; s1, s2, and s3). (a) 16s rDNA abundance. (b) *hgcA* abundance

The copy number of the 16s rDNA and *hgcA* genes in paddy soils at the three mercury mining areas were quantified by qPCR (Figure 2). In our samples, approximately 1% of microbes carried the *hgcA* gene. Different distribution patterns were observed with respect to the *hgcA* abundance at sampling sites, and the pattern was similar to the distribution of total bacterial abundance. In the Wanshan mercury mining area, the *hgcA* gene abundance was negatively correlated with OM (τ = −0.518, *p *<* *.05); in the Fenghuang mercury mining area, the *hgcA* gene abundance was positively correlated with the OM (τ = 0.681, *p *<* *.01) and total mercury content (τ = 0.710, *p *<* *.01); and in the Xunyang mercury mining area, the *hgcA* gene abundance was negatively correlated with pH (τ = −0.947, *p *<* *.01). The observed *hgcA* abundance in the three mercury mining areas did not exhibit an obvious positive correlation with MeHg concentrations, which is not consistent with a previous study (Du et al., 2017) that observed a significant positive correlation between *hgcA* abundance and MeHg concentrations. Although a correlation between the presence of the *hgcA* in the genome of a particular phylogenetic cluster and methylation ability has been demonstrated in the laboratory (Gilmour et al., 2013), no relationships between *hgcA* expression levels and net mercury methylation have been observed (Bravo et al., 2016; Goñi‐Urriza et al., 2015). In particular, the MeHg content in sediments resulted in the increased formation of mercury–OM complexes than did net mercury methylation, which could explain the lack of a significant correlation between *hgcA* gene expression levels and MeHg concentrations. This dual role of degraded OM in the complexation and reduction of Hg can significantly affect the mercury transformation and biological uptake that results in the formation of MeHg (Gu et al., 2011). These facts may explain the negative correlation observed between the OM and MeHg concentrations in mercury mining areas. The pH was also a contributing factor, but only in the Xunyang mercury mining area, which has soil that is partially alkaline. An alkaline environment may be not suitable for the survival of *hgcA* gene containing Hg‐methylating microbes. It is interesting that in the same mercury mining area, the environmental factors that affected the *hgcA* gene abundance were fundamentally different from the environmental factors that affected the MeHg content. Thus, it is difficult to assess the specific effect of a single environmental factor on *hgcA* abundance.

### 3.3 Phylogenetic analyses

**Figure 3:**
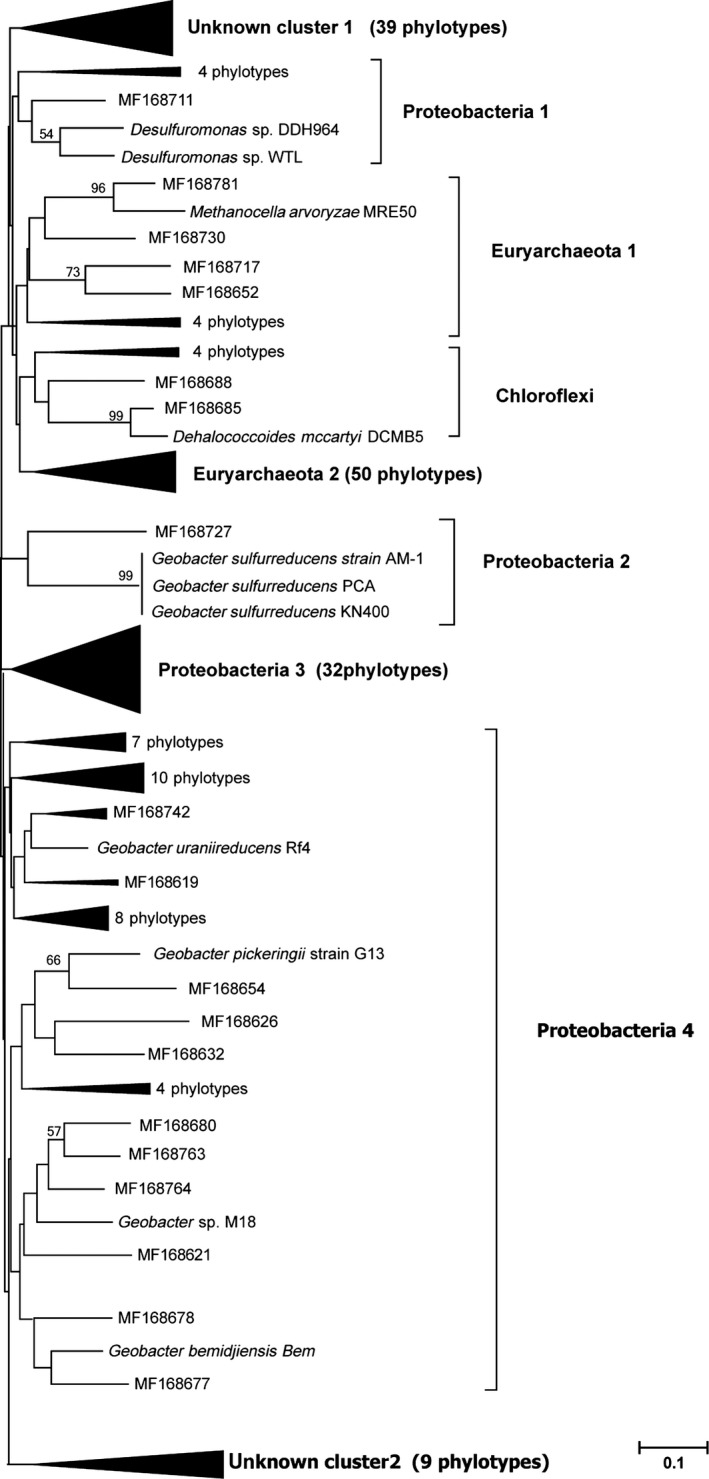
Phylogenetic trees reconstruction from *hgcA* sequences obtained from paddy soils and neighbor‐joining trees based on the Kimura two‐parameter distance with 1,000 replicates to generate bootstrap values using MEGA 6.0. The clones are from g1, g2, g3, h1, h2, h3, s1, s2, and s3, respectively. Bootstrap values (>50%) are indicated at branch points. The scale bar represents a 10% estimated sequence divergence

**Figure 4:**
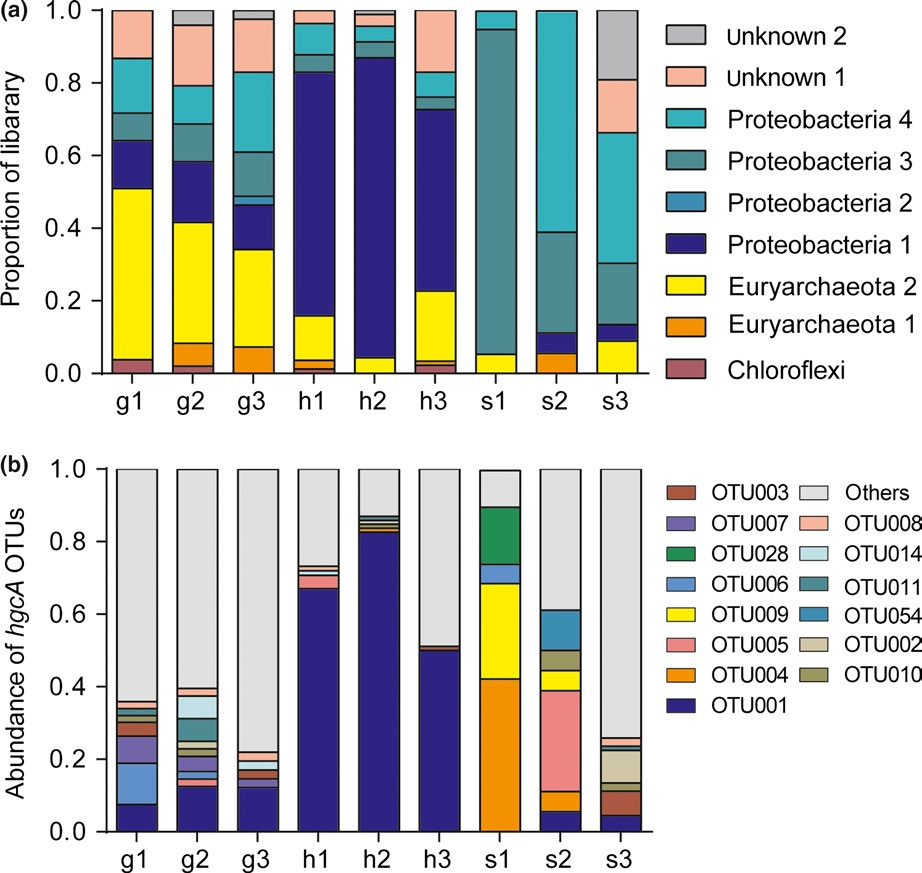
Relative abundances of *hgcA*‐containing microbes at the phylum level in paddy soils. (a) Relative abundances are based on the proportional frequencies of the *hgcA* gene that could be classified at the phylum level. (b) Relative abundances of *hgcA* operational taxonomic units in sampling sites

A phylogenetic tree was reconstructed using the *hgcA* gene sequences obtained from paddy soils and those deposited in the NCBI database (Figure 3). The phylogenetic tree revealed that the *hgcA* gene sequences were divided into eight distinct clusters at the phylum level, including the phyla Proteobacteria (four subclusters), Euryarchaeota (two subclusters), Chloroflexi, and two unnamed clusters. All Proteobacteria clusters belonged to class Deltaproteobacteria, which contains most of the currently confirmed Hg‐methylating microbes. The majority of *hgcA*‐containing microbes in our samples were Deltaproteobacteria, although the distribution patterns in the nine sites were different (Figure 4). In the Guizhou mercury mining area (g1, g2, and g3), the majority of *hgcA*‐containing microbes at the phylum level belonged to Euryarchaeota2, and the Euryarchaeota‐like sequences were closely related to methanogens. At the phylum level, the majority of *hgcA*‐containing microbes in the Hunan mercury mining area (h1, h2, and h3) were Proteobacteria1, and the Proteobacteria1‐like sequences were closely related to SRB. Proteobacteria3 and Proteobacteria4 were the majority in the Shanxi mercury mining area (s1, s2, and s3), and Proteobacteria4‐like sequences were closely related to IRB. In other words, the majority of *hgcA*‐containing microbes were related to Hg‐methylating microbes. However, in the Fenghuang mercury mining area, the total mercury content was close to the local background levels, and the sulfate concentration was not high enough to stimulate the activity of SRB compared to other sites (Zhao et al., 2016). Conversely, due to gene loss and horizontal gene transfer (HGT), gene trees based on single‐function genes sometimes do not represent true species trees (Degnan & Rosenberg, 2006; Song, Liu, Edwards, & Wu, 2012). HGT appears to occur more often within the human microbiome than with unassociated microbes, and seems to be driven by common ecology rather than the phylogenetic distance among species (Koskella, Hall, & Metcalf, 2017). The disconnect between function and species had been frequently reported, because prokaryotes and some eukaryotes are asexual, which is believed to limit speciation. Instead, bacteria form ecological species, which describes a species as a group of individuals who could be considered to be same in relevant ecological properties (Prosser et al., 2007). A previous study demonstrated that amplification of *hgcA* may help establish a link between Hg‐methylating microbes and potential MeHg pollution (Liu, Yu, Zheng, & He, 2014), although the types and ways in which environmental factors affect the distribution patterns of these microbes remain unknown.

### 3.4 Diversity of *hgcA* genes

**Figure 5:**
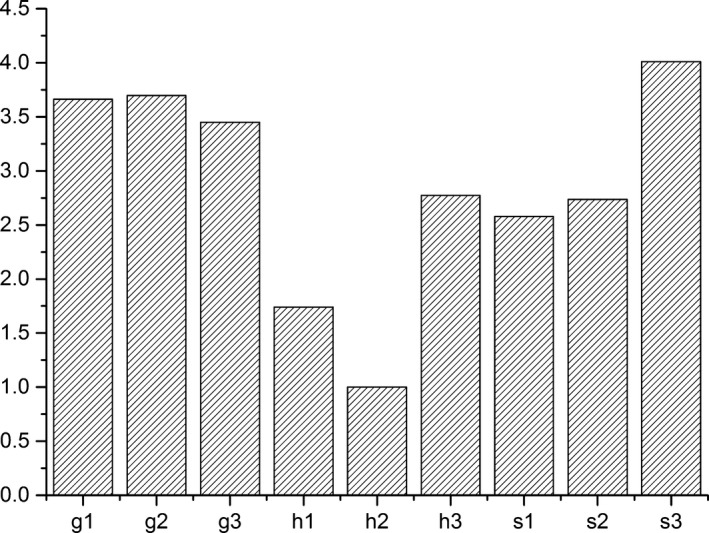
The *hgcA* microbial diversity measured by Shannon–Wiener index in paddy soils

The Shannon index of the obtained *hgcA* genes at the Wanshan mercury mining area is shown in Figure 5. This result showed the greater diversity of the microbial community at the Wanshan mercury mining area (except s3) compared to the other study sites. Previous studies indicated that the total mercury concentration in soils was negatively correlated with the microbial community in situ (Harris‐Hellal, Vallaeys, Garnier‐Zarli, & Bousserrhine, 2009). However, there was no obvious linear correlation between the total mercury content in soil and the α diversity of the microbial community containing *hgcA*. This may result from the complex effect of different variables in paddy soils.

**Figure 6:**
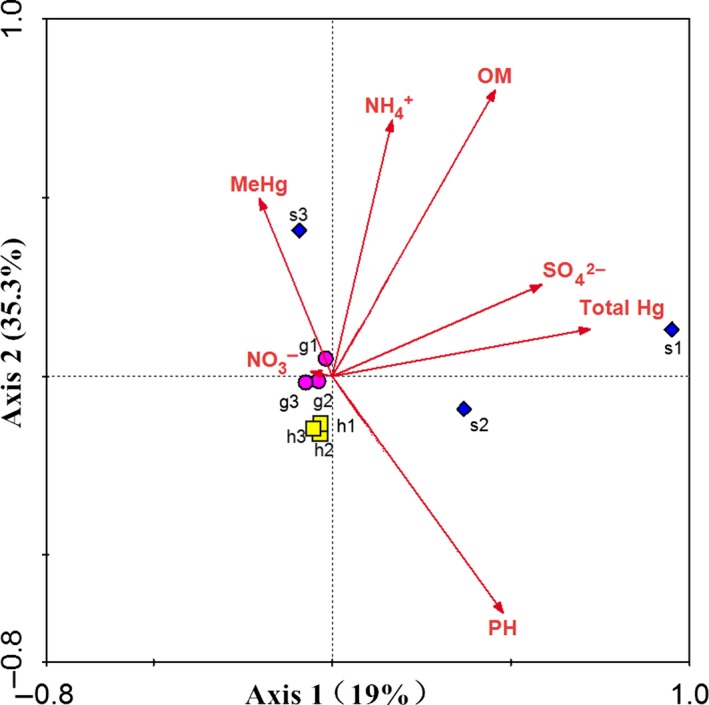
Canonical correlation analysis of relationships between *hgcA*‐containing microbes and environmental factors in soils, including the content of OM, SO_4_^2−^, NH_4_^+^, NO_3_^−^, total Hg, and pH. Values on axes represent cumulative percent variations of sample–environment relationships

**Figure 7:**
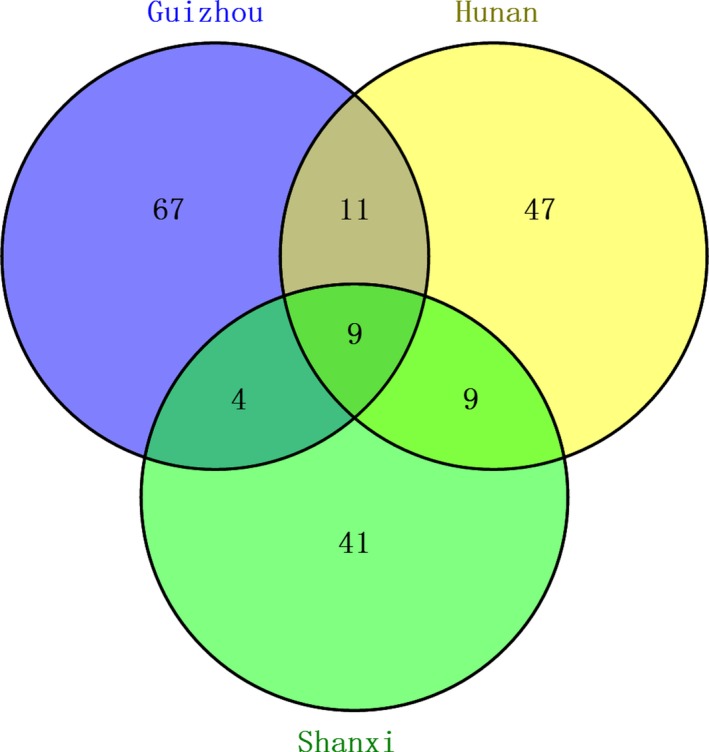
A Venn diagram representing common *hgcA* OTUs. Different colors represent the OTUs from different Hg mining areas. Common OTUs are interconnected between relevant circles. OUTs, operational taxonomic units

The results of the CCA described the effects of environment factors on microbial communities based on *hgcA* (Figure 6). In total, 54.3% of the cumulative percent variance contributed to the relationship of OTUs and the environment by two axes. The chi‐square distance of Guizhou (g1, g2, and g3) and Hunan (h1, h2, and h3) was short, indicating little difference in microbial community composition. However, nine OTUs were shared among all sites, as shown in the Venn diagram of OTUs based on *hgcA* (Figure 7). The number of OTUs shared among Guizhou and Hunan datasets was only 20, which is close to the number shared by the Hunan and Shanxi datasets. Furthermore, the dominant bacteria in the Hunan and Shanxi datasets belonged to Proteobacteria, while the dominant bacteria in Guizhou belonged to Euryarchaeota. The most important variable that impacted the microbial community was pH, possibly because of the effects of pH on the mercury methylation rate. Under acidic conditions, the methylation of mercury at the interface between water and sediments is enhanced, but is inhibited in sediments under anaerobic conditions (Ullrich, Tanton, & Abdrashitova, 2001). The effect of pH on mercury methylation in paddy soils might be similar to that of sediments. In addition, pH can directly affect the solubility of mercury in the soil. When the soil was under acidic conditions, the degree of mercury methylation in the soil was enhanced, resulting in the increased bioavailability of mercury. However, when the pH is too low, a large amount of humic acid could affect the bioavailability of the mercury or the transfer of a methyl group from a methyl donor, decreasing the methylation of mercury. The OM content was the second most significant variable, followed by the content of total mercury, NH_4_^+^, and SO_4_^2−^. OM may play an important role in the complexation of mercury under anoxic conditions and could be expected to significantly affect the production and bioaccumulation of methylmercury (Chiasson‐Gould, Blais, & Poulain, 2014). However, OM is a key driver of Hg reactivity and bioavailability in marine seawater (Schartup, Ndu, Balcom, Mason, & Sunderland, 2015). Recently, Zhao et al., (2017) observed that the effect of OM on Hg methylation was bacterial strain specific. In the current study, the observed influence of total mercury on bacterial community structure might have been due to the high mercury tolerance and resistance of Hg‐methylating microbes (Hoque & Fritscher, 2016). NH_4_^+^ levels may increase ammonium accumulation under anaerobic conditions, which indirectly affects the OM content (Liu, Zheng, Zhang, & He, 2014). Sulfate can enhance the activity of sulfate‐reducing bacteria to enhance the methylation of mercury. Furthermore, sulfide, a metabolite of sulfate‐reducing bacteria, was observed to be continuously combined with mercury to form mercuric sulfide, reducing the bioavailability of mercury and leading to the inhibition of mercury methylation (Zhao et al., 2016).

## 4 CONCLUSIONS

In this study, the responses of microbes containing the *hgcA* gene that were exposed to long‐term mercury contamination were investigated in paddy soils nearby typical mercury mining areas. The soil bacterial community in three types of mercury mines was different, especially the bacterial distribution and composition. However, with respect to soil properties, the soil pH and OM were the main factors influencing the bacterial communities. Thus, microbial communities and various environmental factors should be considered together when assessing the human health risks of paddy soil mercury contamination through the food chain.

## CONFLICT OF INTERESTS

The authors declare that they have no conflicts of interest.
